# Long-Term Outcomes of Sacral Neuromodulation for Refractory Interstitial Cystitis/Bladder Pain Syndrome: A Retrospective Cohort Study

**DOI:** 10.3390/jcm14113647

**Published:** 2025-05-22

**Authors:** Martina Rekatsina, Matteo Luigi Giuseppe Leoni, Veerle Visser-Vandewalle, Marco Mercieri, Giustino Varrassi, Georgios Matis

**Affiliations:** 1 Aretaieion University Hospital, National and Kapodistrian University of Athens, 115 27 Athens, Greece; 2 Department of Medical and Surgical Sciences and Translational Medicine, Sapienza University of Rome, 00185 Rome, Italy; matteoluigigiuseppe.leoni@uniroma1.it (M.L.G.L.); marco.mercieri@uniroma1.it (M.M.); 3 Department of Stereotactic and Functional Neurosurgery, Faculty of Medicine, University Hospital of Cologne, University of Cologne, 50937 Cologne, Germany; veerle.visser-vandewalle@uk-koeln.de (V.V.-V.); georgios.matis@uk-koeln.de (G.M.); 4 Fondazione Paolo Procacci, 00193 Rome, Italy; giuvarr@gmail.com

**Keywords:** Sacral neuromodulation (SNM), interstitial cystitis (IC), bladder pain syndrome (BPS), chronic pelvic pain, refractory IC/BPS, urinary symptoms

## Abstract

**Background**: Interstitial cystitis/bladder pain syndrome (IC/BPS) is a chronic condition characterized by persistent bladder-related pain and urinary symptoms, often refractory to conventional treatments. Sacral neuromodulation (SNM) has emerged as a promising therapeutic option for managing refractory IC/BPS. **Methods**: This retrospective study included 24 patients with IC/BPS treated with SNM between 2017 and 2022. Baseline and follow-up data were collected on pain, opioid use, urinary symptoms, and quality of life. Patients underwent a trial of tonic stimulation before permanent implantation. Continuous variables were reported as median (IQR) and categorical data as counts and percentages. Pre- and post-SNM differences were analyzed using the Wilcoxon rank-sum test. Kaplan–Meier analysis evaluated lead survival, and a Sankey diagram illustrated employment status transitions. **Results**: Patients had a median age of 54.5 years (IQR: 47–61), with 92% female. Subtypes included Type 1 IC/BPS (8.3%), Type 2 (45.8%), Type 3 (37.6%), and unknown type (8.3%). Median pain duration was 4.5 years (IQR: 3–7.3). SNM resulted in significant improvements in pain (NRS: baseline 8 [IQR: 8–9], last follow-up 3 [IQR: 2–4], *p* < 0.0001), opioid use (MME: baseline 20 [IQR: 10–40], last follow-up 0 [IQR: 0–10], *p* < 0.0001), urinary function (24-h voids: baseline 19 [IQR: 14.5–25.8], last follow-up 8 [IQR: 6–12], *p* < 0.0001), and quality of life (QOL) (EQ-5D-5L: baseline 0.50 [IQR: 0.36–0.56], last follow-up 0.83 [IQR: 0.76–0.89], *p* < 0.0001). Employment rates increased from 43.5% to 50%, and unemployment decreased from 8.7% to 4.2%. The median follow-up was 35 months (IQR: 28–53). **Conclusions**: SNM significantly improved pain, urinary symptoms, quality of life, and employment outcomes in patients with refractory IC/BPS. These findings highlight its efficacy as a minimally invasive and reversible option for managing this challenging condition.

## 1. Introduction

Interstitial cystitis or bladder pain syndrome (IC/BPS) is an unpleasant persistent or recurrent chronic sensation that involves pain, pressure, and discomfort perceived to be related to the urinary bladder. It is associated with lower urinary tract symptoms of more than six weeks duration, in the absence of infection or other identifiable causes [1]. This definition was proposed by the Society for Urodynamics and Female Urology (SUFU) in 2009 [2]; however, there is a disagreement between guidelines on the exact definition and the nomenclature to use to describe this condition [3]. Typical symptoms include urinary frequency, nocturia, dysuria, and suprapubic or perineal pain, which may persist even after voiding. Incontinence is not a usual symptom [4]. A rough prevalence estimation for worldwide patients diagnosed with IC/BPS would be about 300 per 100.000 women, while the male prevalence is considered to be 10% to 20% of the female estimate [1]. The lower prevalence in men may be attributed to both under-reporting and overlap with chronic prostatitis/chronic pelvic pain syndrome [5]. It is most often diagnosed in the fourth decade or later [4,6]. The diagnosis of IC/BPS is based on history, physical examination, and laboratory testing that should be conducted both to confirm the presence of symptoms characteristic of IC/BPS and to rule out other conditions [4]. IC/BPS aetiology is under continuous investigation. Immune cell activation, increased urothelium permeability, inhibition of bladder urothelial cell proliferation, autoimmune mechanisms, the bladder’s reaction to infection, neurobiological mechanisms, pelvic organ crosstalk, toxic agents in the urine, relative bladder hypoxia, and genetic predisposition have been suggested as possible mechanisms [1]. A Hunner’s lesion (or Hunner lesion) is observed in 5–57% of IC cases and appears as a characteristic inflammatory response [7]. It is typically seen as a reddened area of mucosa with small blood vessels converging towards a central scar. During bladder distension, these vessels often split, leading to a distinct waterfall-like bleeding pattern [7]. Hunner Lesion Disease (HLD) has started to be considered as a distinct disease and is managed accordingly [1], as it differs in treatment and outcome [8]. Guidelines for its management have been issued by both the American Urological Association (AUA) and Canadian Urological Association (CUA) [4,9]. The classification of IC and IC-related conditions differs among guidelines. The AUA Guidelines use IC/BPS as a single term combining all presumed classifications without further sub-classification. The ESSIC (International Society for the Study of Bladder Pain Syndrome) uses BPS as the umbrella term for this condition and divides it into Type 1, Type 2, or Type 3, depending on the presence or absence of Hunner lesions and/or glomerulations [10]. The East Asian Guidelines have no umbrella term but three categories: Hunner type IC, non-Hunner type IC, and hypersensitive bladder syndrome, according to the presence or absence of Hunner lesions and/or glomerulations [11,12,13].

Controlling pain and other symptoms in IC/BPS can be challenging. First-line management of non-Hunner lesion IC/BPS includes patient education, stress reduction, dietary changes, over-the-counter analgesics, as well as pelvic floor relaxation and physical therapy. If these measures fail, other oral (tricyclic antidepressants, antihistamines, immunosuppressants, permeability regulators, sildenafil, quercetin, palmitoylethanolamide) and intravesical therapies along with cystoscopy may follow as second line. Hunner lesion disease, however, requires additional interventions to reduce lesions. More advanced approaches, such as intra-mural botulinum toxin (BTX-A) and neuromodulation (sacral nerve stimulation), despite being third-line options, can be offered if symptoms persist [1]. Both CUA and AUA support the use of neuromodulation for refractory cases [4,9].

### Role of Sacral Neuromodulation in IC/BPS and Mechanisms of Action

Sacral neuromodulation (SNM) has been shown in most studies to be a reasonably effective treatment for controlling intractable bladder overactivity and chronic pelvic pain in patients who have not achieved adequate symptom control with other modalities [14,15,16,17,18]. It is a promising treatment modality for refractory IC/BPS, especially due to its minimally invasive nature, and it should be tried before rigorous surgery [19]. Neuromodulation is a physiological process in which activity within one neural pathway modulates pre-existing activity in another pathway through synaptic interactions [20]. SNM regulates excitatory and inhibitory impulses affecting pelvic organs by stimulating pelvic afferent nerves. It influences spinal reflexes and brain networks, with studies showing decreased brain activity in regions like the anterior cingulate cortex and prefrontal cortex in overactive bladder patients after SNM. Brain activity also changes depending on SNM stimulus intensity [21,22]. SNM’s effects on pain align with the gate control theory by Melzack and Wall, suggesting that non-nociceptive impulses can inhibit visceral pain at the spinal level [23]. It also modulates pain via descending pathways from the brainstem and limbic system [20]. Research has shown overlapping spinal regions process nociceptive inputs from the bladder and urethra, supporting Ruch’s convergence theory, which explains visceral referred pain as shared central pathways for visceral and somatic nociceptive signals [24]. It was also demonstrated that specific spinal regions, including the dorsal commissure and lateral laminae near the sacral parasympathetic nucleus, exhibit increased c-fos expression in response to both noxious and non-noxious bladder inputs, converging on the same dermatome [25].

This study aims to present the long-term experience of the Department of Stereotactic and Functional Neurosurgery, Faculty of Medicine and University Hospital of Cologne, University of Cologne, Cologne, Germany, in managing patients with IC/BPS and refractory pain.

## 2. Materials and Methods

**Figure 1:**
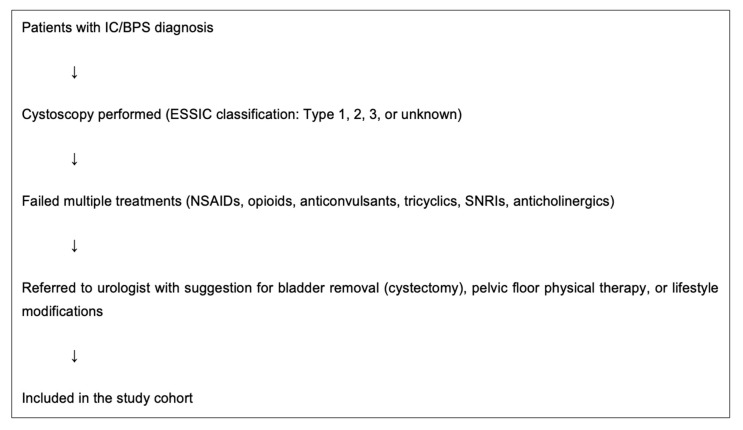
Patient inclusion process.

This is a single center retrospective cohort study conducted in the Department of Stereotactic and Functional Neurosurgery, Faculty of Medicine and University Hospital of Cologne, University of Cologne, Cologne, Germany, utilizing patients’ medical records. The study included patients with IC/BPS that, beyond having already tried several medications (NSAIDs, opioids, anticonvulsants, tricyclics, SNRIs, anticholinergic), had also visited urologist(s) who could offer only a removal of the bladder (cystectomy), pelvic floor physical therapy, and lifestyle changes. All patients had had a cystoscopy before their initial visit to our service and were categorized according to the ESSIC subtypes of IC/BPS: Type 1 (normal bladder wall), Type 2 (glomerulations), Type 3 (Hunner’s lesions), and unknown type [10]. The study included patients diagnosed with IC/BPS who had previously undergone cystoscopy (ESSIC classification Types 1–3 and unknown), had failed multiple pharmacological treatments (NSAIDs, opioids, anticonvulsants, tricyclics, SNRIs, anticholinergics), and had been recommended for cystectomy, pelvic floor physiotherapy, or lifestyle modifications by urologists. Patients without a prior cystoscopy or without documentation of prior therapeutic failures were excluded (Figure 1).

The procedure involved implantation of 1 or 2 leads with 8 contacts via the sacral hiatus. We offered a 10-day trial with stimulation of S2, S3, and S4 roots (tonic stimulation; settings: frequency: 14–20 Hz, pulse width: 180–240 µs, amplitudes: lightly below the sensory threshold (70% of the threshold). Systems implanted: Precision Novi^TM^, Precision Montage^TM^, Precision Spectra™, Spectra WaveWriter™, WaveWriter Alpha™ (Boston Scientific, Valencia, CA, USA). The trial was deemed successful if patients experienced at least a 50% reduction in pain, after which they were scheduled for permanent system implantation within two weeks. The implantable pulse generator (IPG) was consequently placed in the gluteal region. When it was technically possible, we strived to implant 2 leads in order to achieve better pain coverage. If it was not technically feasible and to minimize the risk of surgical complications, we implanted only 1 lead.

**Figure 2:**
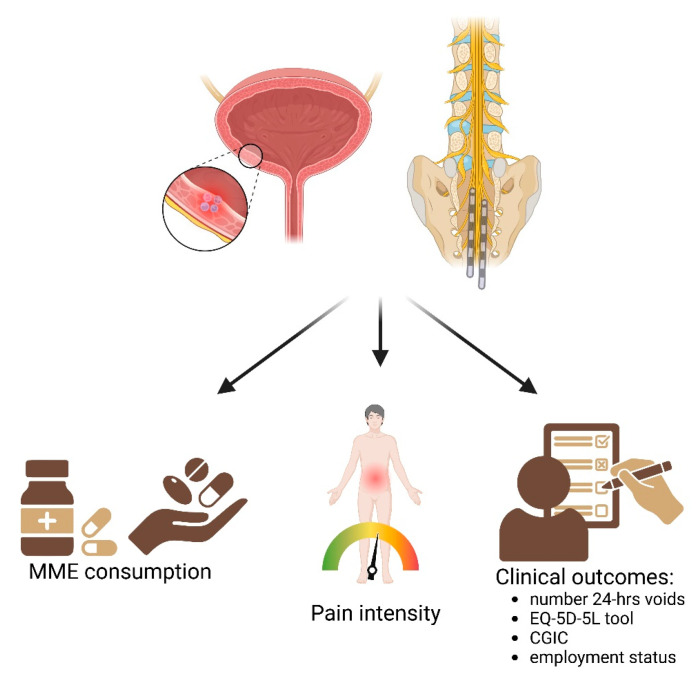
Graphical representation of assessed parameters following SNS for IC/BPS. The procedure involved the implantation of one or two leads via the sacral hiatus, followed by a trial period to evaluate efficacy in pain relief. Key outcomes measured include daily morphine milligram equivalents (MME) consumption, pain intensity (NRS scale), and clinical outcomes such as the number of 24 h voids, health-related quality of life (EQ-5D-5L), Clinical Global Impression of Change (CGIC), and changes in employment status.

The following parameters were assessed: changes in daily morphine milligram equivalents (MME) consumption, pain intensity (measured on the NRS scale), the number of 24 h voids, health-related quality of life (HR-QOL) using the EQ-5D-5L tool, the Clinical Global Impression of Change (CGIC), and changes in employment status (Figure 2). The data presented were collected on the last follow-up visit.

### Data Analysis

Continuous variables were presented as median values with interquartile ranges, while categorical data were expressed as absolute numbers and percentages. The Shapiro–Wilk test was applied to assess data distribution. Differences in continuous variables before and after SNM were analyzed using the Wilcoxon rank-sum test. A Kaplan–Meier survival curve was employed to illustrate lead survival probabilities over time following implantation. A Sankey diagram was used to visualize participants’ employment status transitions, highlighting changes between baseline and post-SNM follow-up. Statistical significance was set at a two-tailed *p* value < 0.05. All analyses were conducted using R version 4.2.2 (R Foundation for Statistical Computing, Vienna, Austria; www.r-project.org, accessed on 15 March 2025)

## 3. Results

We included 24 patients with IC/BPS who were treated in the Department of Stereotactic and Functional Neurosurgery, Faculty of Medicine and University Hospital of Cologne, University of Cologne, Cologne, Germany between January 2017 and December 2022. The cohort had a median age of 54.5 years (IQR: 47–61), with the majority being female (92%) and only 8% male. Subtype distribution revealed that 8.3% of patients had Type 1 IC/BPS (normal bladder wall), 45.8% had Type 2 (glomerulations), 37.6% had Type 3 (Hunner’s lesions), while 8.3% had an unknown subtype. The median duration of pain was 4.5 years (IQR: 3–7.3). Comorbidities were common, with 33.3% of patients reporting irritable bowel syndrome, 25% migraine, 41.7% bacterial cystitis, 16.7% rheumatoid arthritis, and 16.7% fibromyalgia, (Table 1).

**Figure 3:**
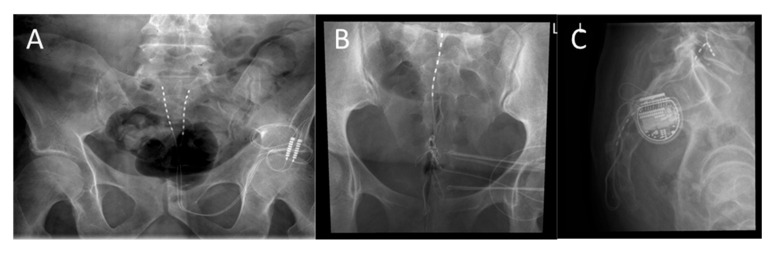
(**A**) 55-year-old female patient with IC, 2 linear leads with 8 contacts (4 mm spacing), (**B**) 60-year-old female patient with IC, 1 linear lead with 8 contacts (4 mm spacing). (**C**) 62-year-old male patient with IC, 2 linear leads with 8 contacts (4 mm spacing), dislocation of 1 lead cranially.

Eighteen patients (75%) were implanted with two leads, each with eight contacts spaced 4 mm apart (Figure 3A), while six patients (25%) received a single lead with eight contacts and the same 4 mm spacing (Figure 3B). One patient (4.2%) required surgical revision 28 months post-implantation due to cranial lead dislocation (Figure 3C).

**Figure 4:**
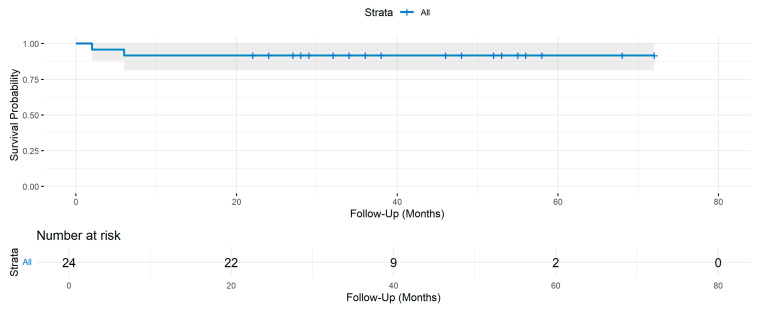
Kaplan–Meier survival curve showing the probability of lead survival over time in months following implantation. The shaded area represents the 95% confidence interval. The number of patients at risk is displayed below the graph at different time points.

Two patients underwent explantation for different reasons: one was explanted after 2 months due to dissatisfaction from inadequate pain relief, and the other was explanted after 2 months due to a localized infection at the site of lead anchoring (Figure 4). The median follow-up duration following implantation was 35 months (IQR: 28–53 months).

**Figure 5:**
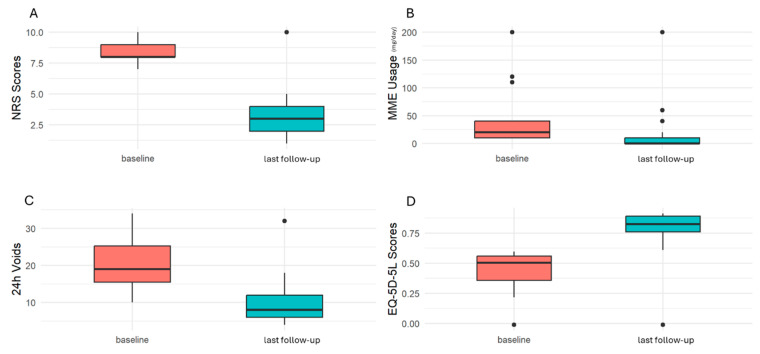
Changes observed in key clinical parameters before and after SNM. Data are reported as box plots indicating the median and interquartile range (IQR). Points outside the whiskers represent outliers. (**A**). NRS Scores before and after SNM, (**B**) Morphine Equivalent usage before and after SNM, (**C**) 24 h urinary function before and after SNM, (**D**) EQ-5D-5L Scores before and after SNM.

A significant pain reduction was observed at last follow-up compared to pre-SNM (NRS baseline 8 (IQR: 8–9), NRS last follow-up 3 (IQR: 2–4), *p* < 0.0001), with a median reduction of 60% (Figure 5A). Along with pain reduction, a significant decrease in MME usage was observed (MME baseline 20 (IQR: 10–40), MME last follow-up 0 (IQR: 0-10), *p* < 0.0001), (Figure 5B). Interestingly, following SNM implantation, 13 patients (54%) were able to discontinue opioid use entirely. A significant reduction in the number of 24 h voids was observed from baseline to the last follow-up (24 h voids baseline 19 (IQR: 14.5–25.8), 24 h voids last follow-up 8 (IQR: 6–12), *p* < 0.0001), with a median reduction of 56%, indicating a marked improvement in urinary function (Figure 5C). All the observed improvements led to a significant enhancement in EQ-5D-5L scores, reflecting improved health-related QOL during the follow-up period (EQ-5D-5L baseline 0.50 (IQR: 0.36–0.56), EQ-5D-5L last follow-up 0.83 (IQR: 0.76–0.89), *p* < 0.0001), with a median improvement of 61% (Figure 5D).

**Figure 6:**
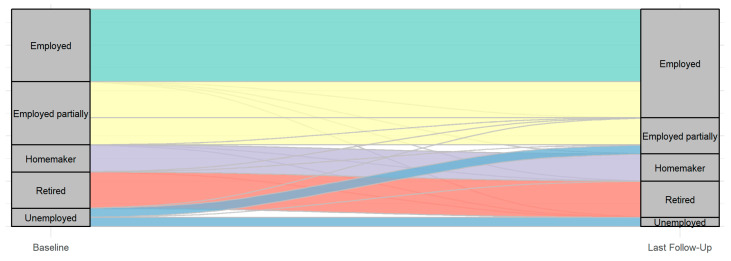
Changes in the employment status among participants prior to SNM and at the last follow-up.

Eighteen patients (75%) reported a substantial improvement in their condition following SNM, five patients (20%) reported moderate improvement, and one patient (5%) reported no improvement, as assessed by the CGIC score. The changes in employment status among participants before SNM and at the last follow-up are shown in Figure 6. At baseline, the majority of participants were employed (43.5%) or employed partially (26.1%), while smaller proportions were homemakers (4.4%), retired (17.4%), or unemployed (8.7%). By the last follow-up, the proportion of employed participants increased to 50%, suggesting that SNM may have contributed to enhancing and maintaining participants’ work capabilities. A slight decrease was observed in the employed partially group (16.7%), as three patients (12.5%) transitioned from partial to full employment following SNM, and one participant (4.2%) who was previously unemployed became partially employed. Consequently, the percentage of participants in the unemployed group decreased to 4.2% at the last follow-up following SNM. This shift indicated a potential improvement in workforce participation. The homemaker and retired groups remained unchanged. These findings suggest that sacral stimulation may positively influence participants’ ability to maintain or improve their employment status, thereby contributing to enhanced functional outcomes and quality of life.

## 4. Discussion

The results of this study highlight the significant benefits of SNM in managing IC/BPS. The treatment was associated with substantial improvements in key clinical outcomes, including pain intensity, MME consumption, urinary function, and overall quality of life.

IC/BPS is a debilitating condition affecting a significant number of individuals worldwide [1]. Various treatments strategies have been suggested according to the AUA and CUAJ [4,9]. The AUA suggests a stepwise approach, starting with behavioral and non-pharmacologic treatments, followed by oral medications, intravesical instillations, cystoscopy with hydrodistension, and BTX-A injections as intermediate steps before considering major surgical interventions [4,9]. In contrast, the CUAJ proposes symptom-specific management based on a detailed phenotyping approach that addresses urinary, psychosocial, organ-specific, infectious, neurologic/systemic, and tenderness-related factors [4,9]. SNM is recognized by both associations as a viable option for managing refractory IC/BPS, classified as a Grade C recommendation. This minimally invasive option should be offered to patients who have symptoms refractory to multiple other treatments after a successful trial [4,9]. Compared to the above-mentioned treatment modalities, SNM offers an alternative for patients who have exhausted conservative therapies and are at risk of requiring major surgical interventions, providing a potential means to improve outcomes before resorting to cystectomy.

While comprehensive systematic reviews and meta-analyses up to 2017 have highlighted the efficacy and safety of SNM in treating refractory IC/BPS [16], there remains a significant lack of more recent large-scale randomized controlled trials (RCTs) dedicated specifically to this therapeutic modality. The meta-analysis by Wang et al. included 17 studies encompassing 583 patients and demonstrated significant improvements in pelvic pain, urinary frequency, and urgency, with a pooled treatment success rate of 84%. This analysis provided robust evidence supporting SNM as both an effective and safe intervention for refractory IC/BPS. Despite subsequent advancements in neuromodulation techniques and device technologies, high-quality studies focusing solely on SNM for IC/BPS remain limited.

In our study, we chose to insert the electrodes via the caudal route to increase safety and ease implantation procedure. Previous studies have described alternative approaches, including foraminal placement or open access techniques [26,27,28].

Feler et al. published a case series of sacral SNM in patients with pelvic pain, using a rostro-caudal method targeting the S2, S3, and S4 roots with four quadripolar leads. If the foraminal route was deemed unfeasible due to adhesions or technical challenges, an open implantation of a pair of plate electrodes was performed [29]. Their findings suggested that the intraforaminal method might increase the risk of complications, such as inadvertent intrathecal implantation [29].

Zermann et al. reported a case study of a female patient who had been suffering from painful IC for more than five years and was successfully treated with SNM [30]. During the trial phase, stimulation of the S3 sacral nerve with a wire electrode resulted in a reduction in micturition frequency, significant rectal pain relief, and the absence of pain trigger points within the first 48 h. Following the successful trial, the patient underwent implantation of a permanent SNM system [30].

Our results confirm the long-term (>4 years) effectiveness of SNM. In fact, our patients had a significant pain reduction from baseline (NRS from 8 to 3; median reduction 60%) which was accompanied by a significant reduction or discontinuation of opioids. This high success rate is also confirmed by the study of Kütükoğlu et al., who reported a similar success rate of 58.5% in IC patients [31]. Additionally, the study of Feler et al. reported a 30% reduction in opioid use and a 30% discontinuation rate four months after SNM implantation for pelvic pain [29].

We also showed that SNM significantly improved urinary function by reducing the void episodes by more than 50%. Conversely, a previous study investigating the effectiveness of SNM in patients with lower urinary tract dysfunction of various aetiologies reported a 70% improvement [32]. Although this percentage is higher from our study, the difference could be attributed to the different aetiologies and not just IC/BPS.

As a result of reduced pain scores and improved bladder function, the quality of life (QOL) of our patients significantly improved, with a median enhancement of 61%. Notably, the majority of patients (75%) reported substantial improvement in their condition following SNM. Other studies investigating the impact of SNM across various aetiologies also demonstrated significant QOL improvements, often strongly correlated with a reduction in the number of incontinence episodes [33]. Hernández-Hernández et al. specifically reported a significant improvement in QOL among IC/BPS patients (*p* < 0.0001), (Pre-QOL: 17.86 ± 16.04; Post-QOL: 75.71 ± 24.90) [34]. Another study examining the efficacy of SNM in refractory IC/pelvic pain syndrome revealed statistically significant improvements in voiding frequency, nocturia, average voiding volume, QOL score, and NRS (all *p* < 0.05) [35].

The strengths of our study include the extended follow-up period of 35 months and the low rate of explantation. The discontinuation rate due to inadequate pain relief was notably low, with only 1 patient (4.2%) undergoing explantation out of the initial 24 participants. Similarly, the revision and complication rates were minimal, both at 4.2%. Additionally, we demonstrated a positive impact on employment status, likely resulting from the improved functional outcomes. This finding was further supported by Feler et al., who reported that 30% of patients resumed work following SNM [29].

These findings support the role of SNM as an effective, minimally invasive treatment option for refractory IC/BPS, offering significant improvements in symptom management, functional outcomes, and quality of life for patients who had previously exhausted other noninvasive treatment modalities.

### Limitations

Our study had several limitations that should be acknowledged. Firstly, it was a single-center retrospective study, which inherently limits the generalizability of the findings. Secondly, the relatively small sample size of 24 patients, while providing valuable insights, may not be sufficient to capture the full spectrum of patient outcomes or account for variations in response to SNM. The significant and consistently positive outcomes observed across all patients in our study, while encouraging, limit the ability to develop predictive models of efficacy. In fact, this homogeneity in response prevents us from identifying potential predictors of success or understanding whether individual factors, such as demographic or clinical variables, could influence treatment efficacy. Without variability in outcomes, it is challenging to determine whether certain subgroups of patients might benefit more from SNM or whether specific baseline characteristics could explain the observed improvements. Future studies with larger and more diverse patient populations, including those with varying levels of response, are essential to address this gap and to better tailor SNM to individual needs. Due to the retrospective nature of this study, detailed physiological and clinical information, such as diabetes status, smoking history, and other relevant comorbidities, was not consistently available across all patients. This limits our ability to assess the potential influence of these factors on the outcomes. Additionally, our study did not include a control group for comparison, making it difficult to fully isolate the effects of SNM from potential confounding variables. It is important to highlight that the majority of participants in our study were female. This gender imbalance may reflect the epidemiological characteristics of IC/BPS, where prevalence is higher among women. This potential limitation should be considered when interpreting the findings and highlights the need for further research in male populations. Finally, the long-term follow-up, while a strength, was not uniform across all patients, which may have introduced variability in outcome assessment. Despite these limitations, our findings provide valuable preliminary evidence and underscore the need for more comprehensive studies to validate and expand upon our results. A multicenter trial with a larger and more diverse patient population would help validate our findings.

## 5. Conclusions

IC/BPS is a chronic condition characterized by persistent bladder-related pain and urinary symptoms, often resistant to conventional therapies. SNM has emerged as a promising therapeutic option for managing refractory IC/BPS. In our study, SNM significantly improved pain levels, urinary symptoms, quality of life, and employment outcomes in patients with refractory IC/BPS. These results highlight SNM as an effective, minimally invasive, and reversible treatment approach, offering hope for patients grappling with this challenging and debilitating condition.

## Figures and Tables

**Table 1:** Baseline characteristics of IC/BPS patients. Continuous variables are reported as median (IQR), while categorical variables are presented as numbers with corresponding percentages.

Variable	IC/BPS Group, n = 24, n (%)
Age, median (IQR), years	54.5 (47–61)
Gender	
Male	2 (8%)
Female	22 (92%)
IC/BPS subtype according to biopsy	
Type 1 (normal bladder wall)	2 (8.3%)
Type 2 (glomerulations)	11 (45.8%)
Type 3 (Hunner’s lesions)	9 (37.6%)
Unknown type	2 (8.3%)
Pain duration, median (IQR), years	4.5 (3–7.3)
Comorbidities	
Irritable bowel syndrome	8 (33.3%)
Migraine	6 (25%)
Bacterial cystitis	10 (41.7%)
Rheumatoid arthritis	4 (16.7%)
Fibromyalgia	4 (16.7%)

## Data Availability

The data presented in this study are available on reasonable request from the corresponding author.

## Abbreviations

The following abbreviations are used in this manuscript:

AUA	American Urological Association
BTX-A	Botulinum Toxin A
BPS	Bladder Pain Syndrome
CGIC	Clinical Global Impression of Change
CUA	Canadian Urological Association
CUAJ	*Canadian Urological Association Journal*
EQ-5D-5L	EuroQol Five-Dimension, Five-Level Questionnaire
ESSIC	International Society for the Study of Bladder Pain Syndrome
HLD	Hunner Lesion Disease
IC	Interstitial Cystitis
IC/BPS	Interstitial Cystitis/Bladder Pain Syndrome
MME	Morphine Milligram Equivalent
NRS	Numeric Rating Scale
NSAIDs	Non-Steroidal Anti-Inflammatory Drugs
RCTs	Randomized Controlled Trials
SNM	Sacral Neuromodulation
SUFU	Society for Urodynamics and Female Urology
